# Supplementary figures and images for: Are rapid tests and confirmatory western blot used for cattle and small ruminants TSEs reliable tools for the diagnosis of Chronic Wasting Disease in Europe?
Source: PLoS One. 2023 Aug 30;18(8):e0286266. doi: 10.1371/journal.pone.0286266 (PMC10468065; doi:10.1371/journal.pone.0286266)

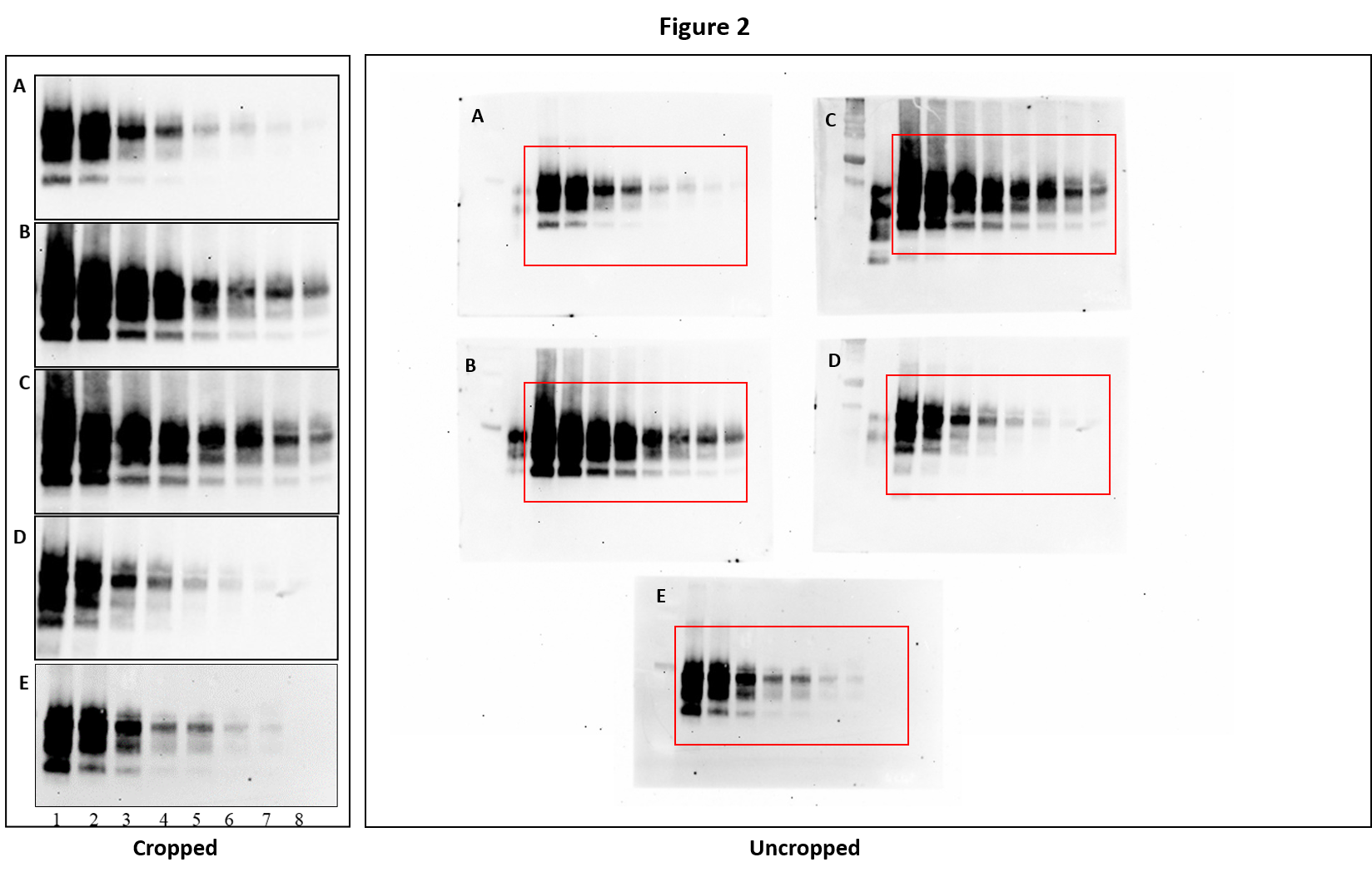

Supplement: S1 Raw image — Confirmatory SAF-Immunoblot analysis of proteinase K-treated homogenates on dilution series from brain tissue of positive Moose D and immunorevealed with five different anti-PrP monoclonal antibodies: 6H4 (A), Sha31 (B), 9A2 (C), SAF84 (D), L42 (E). Lane 1 = undiluted samples; lane 2 to 8 = two-fold dilutions of the sample from 1:2 to 1:128. (TIF) [file pone.0286266.s001.tif]

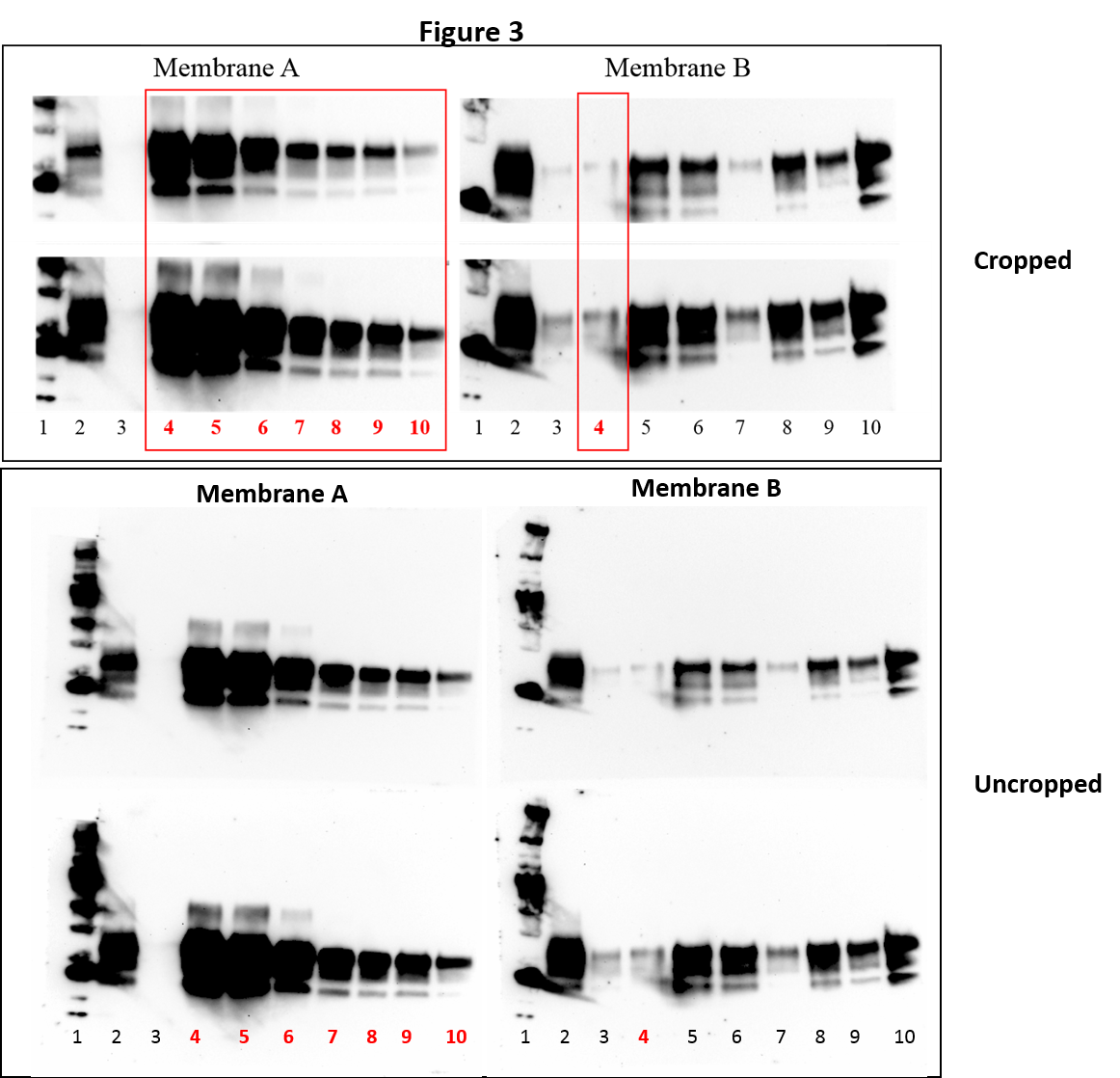

Supplement: S2 Raw image — TeSeE™ Western blot analysis of proteinase K-treated homogenates on dilution series from brain tissue of four positive Moose A, B, C and D. Upper panel short exposition, lower panel longer exposition. Membrane A. Lane 1 = molecular weight; lane 2 = positive classical scrapie; 3 = negative moose control; lane 4 to 10: Moose D; lane 4 = undiluted; lane 5 to 10 = two-fold dilutions of the sample from 1:2 to 1:64. Membrane B. Lane 1 = molecular weight; 2 = positive classical scrapie; lane 3 = Moose E undiluted; lane 4 = Moose D dilution 1:128; lane 5 to 7: Moose A; lane 5 = undiluted; lane 6 = dilution 1:2; lane 7 = dilution 1:4; lane 8 to 9: Moose B; lane 8 = undiluted; lane 9 = dilution 1:2; lane 10 = Moose C undiluted. (TIF) [file pone.0286266.s002.tif]
