# Supplementary material for: Are rapid tests and confirmatory western blot used for cattle and small ruminants TSEs reliable tools for the diagnosis of Chronic Wasting Disease in Europe?
Source: PLoS One. 2023 Aug 30;18(8):e0286266. doi: 10.1371/journal.pone.0286266 (PMC10468065; doi:10.1371/journal.pone.0286266)
Supplement: Attachment — Submitted filename: Response to Reviewers.docx [file pone.0286266.s003.docx]

# Manuscript No. PONE-D-22-31903

**Response to Reviewers**

**Reviewer #1**

| **Query** | **Answer** |
| --- | --- |
| My First question is on the reasons why the authors have not tried all the CWD samples in parallel in the two different laboratories. This might have been more stringent for comparing the reproducibility and the efficiencies of the different diagnostic tests used, especially when different methodologies were used [ie; manual protocol (at IRV) versus robotic system (at NVI) for example]. | The authors agree on the importance of performing all the analyses in parallel in the two laboratories to evaluate the reproducibility of the different diagnostic test, but unfortunately, this was not possible due to the scarcity of material available from these positive cases. Nevertheless, the authors think it is important to provide the scientific field community the data obtained from this study. |
| Line 62: Prion accumulation in the brain leads to neurodegeneration and all the time to death. Why do the authors say « eventually to death » in this sentence? | If the authors are not mistaking, “eventually” in English means “in the end” and should be correct. |
| Lanes 93-96: I Wonder why the Prionics WB test was not mentioned and eventually used in this comparative study. | The authors did not use the Prionics Western Blot as the production of this test has been discontinued since January 2021. Like we mentioned in the Introduction of the manuscript, we used the “three rapid tests that are currently commercially available…..” (lines 92-93). |
| Lines 97-98: What the authors mean by « are not comprehensive in the sentence starting with « Data on the performances… »? | The authors indeed realised that this term is unclear and therefore propose to replace it by exhaustive. |
| Lines 119-120: How many negative animals were used to prepare the negative brain pools of Moose and Reindeer? | Brain material from six moose and six reindeer for set 1 and from 14 moose for set 2 was used to prepare the negative brain pools. The clarification is now written in the text.  All these samples were previously confirmed negative for CWD by confirmatory tests. |
| Line 119: Which type of lymph node was used? | It is the retropharyngeal lymph node, which is the sample recommended along with the brainstem for the surveillance programme of CWD. It is now included in the text. |
| Line 227-228: The O.D. values of undiluted samples that are the only ones detected by the TSE^TM^SAP Combi Kit are not reported on Table 2. Please clarify. | The authors omitted to test undiluted samples because of the paucity of the material. |
| Line 230: As in Table 2 animals are designed by only their corresponding letter, please let this nomenclature in the text (Reindeer 17-CD2788 is Reindeer A). | The authors have identified Reindeer 18-CD2788 as Reindeer A, as suggested. In addition, we discovered an unfortunate typo mistake and corrected from 17-CD2788 to 18-CD2788 in table 1. |
| Lines 239-240: I do not see on the western blot of Figure 3 any difference in PrP^res^ molecular mass between Moose D and the other samples, as claimed by the authors. Please clarify. | The authors agree with this remark, the difference in molecular weight between the moose is not visible on the proposed figure. The TeSeE western Blot is, in our hands, a very efficient test for the detection of PrP^Sc^, extremely useful in the context of confirmatory diagnostic; nevertheless, the test is not ideal to demonstrate a reproducible banding pattern for comparison among TSE isolates, in contrast to the WB method described in Pirisinu et al 2018 (Emerg Infect Dis. 2018; 24(12):2210-2218).  The authors would like to show to Reviewer 1 a better picture of the difference between the moose C and D, in the “Figure to Reviewer 1” under the present table.  As a response to this appropriate remark, we changed the sentence pointed out by the Reviewer to: “In addition, all tests detected easily PrP^Sc^ in moose D which is less terminal truncated than the other moose cases analysed” (lines 255-256). |
| Why the WB blot of the Reindeer samples were not done and were not shown? | The samples from the first set were not analysed by WB, the authors regrettably had to make a choice because of the shortening of quantity of homogenate we had available and decided to run only the different ELISA tests on this first set.  Nevertheless, all the reindeer detected with CWD by ELISA through the surveillance program were confirmed by TeSeE western blot. |
| Lines 268 and 277: In the discussion section, the authors talk about CWD strains. However, at the stage of the present analysis, it might be more appropriate to use the term isolate. | The authors certainly agree that molecular analyses of isolates on their own cannot determine a strain, nevertheless as experimental studies in animals have demonstrated there are different strains, we consider that it is reasonable to call them CWD strains and not isolates.  The references of some of these studies are reported below:  -Nonno et al. 2020. Studies in bank voles reveal strain differences between chronic wasting disease prions from Norway and North America. Proc Natl Acad Sci U S A. Dec 8;117(49):31417-31426. doi: 10.1073/pnas.2013237117.  -Bian et al. Adaptive selection of a prion strain conformer corresponding to established North American CWD during propagation of novel emergent Norwegian strains in mice expressing elk or deer prion protein. 2021. PLoS Pathog.;17(7): e1009748. <https://doi.org/10.1371/journal.ppat.1009748>. |
| Homogenise Proteinase K as PK and not pK | The authors have conformed the definition of Proteinase K as suggested throughout the text. |
| Homogenise g and not G or RCF when describing centrifugation steps. | The authors have conformed the definition of centrifugation steps with “g” as suggested throughout the text. |
| Lines 159: The PK resistance of PrP^Sc^ is not only due to its beta-sheet structure but also to its aggregative state. | The authors have reported in the manuscripts “… due to its beta-sheet structure and to its aggregation”. |
| Line 290: replace than by that? | The authors have replaced than with that, as suggested (line 305). |
| Figure 3: is it necessary to show the two exposure times for WB revelation? The longest one might be enough as it shows all the results. | The authors thank the Reviewer for this suggestion; however they think that the short exposure allows to distinguish the 3 bands better for the strong samples. |

**Reviewer #2**

| **Query** | **Answer** |
| --- | --- |
| The introduction part concerning the work done is a bit misleading. The authors state “performances of these tests were compared to two confirmatory western blot methods…”. However, Fig 1 does not show that set 1 has been tested using TeSeE western blot and confirmatory SAF-immunoblot but another test HerdChek CWD Antigen Test has been used. This latter test is not presented in the materials and methods section. It has also not been tested on set 2. Could the authors comment this point? Have they performed the confirmatory tests they present on set 1? | The authors agree that it can be confusing for the reader that the two sets of samples were not analysed with the same methods. This is due to the paucity of positive cervid tissues, the authors could not go through a complete parallel study in both laboratories, which had been ideal.  The precision has been written in the text, line 105-112.  Set 1 samples were confirmed positive with the TeSeE Western blot routinely used in the Norwegian laboratory following a positive result in the rapid screening test.  Thanks to the comment of the Reviewer, we noticed that we forgot to include the HerdChek CWD Antigen test in the materials and methods section, and it is now included. |
| Among the 41 CWD cases reported in Scandinavia, in reindeer, moose and red deer, 7 infected animals were chosen as positive controls. Could the authors explain their choice? Assessing the sensitivity of the tests initially designed for TSE detection in small ruminants and cattle, would require as many positive samples as possible. | The authors totally agree with this comment. Unfortunately, the authors could only include in the study samples for which enough material was available. Nevertheless, even if the number of animals can be considered small, this is to date the only material available to provide the scientific field community information on rapid tests performance for CWD surveillance. |
| Sensitivity is an important parameter in diagnosis tests, however specificity also. The authors never really mentioned this point in their manuscript. Could they make any comment and evaluate this parameter? | The authors completely agree with the Reviewer’s observation; availability of larger amount of positive and negative material would have allowed the accuracy of these tests based on diagnostic sensitivity and specificity. Nevertheless, based on the paucity of material available the authors choose to focus the study on the analytical sensitivity of the tests as far as possible. |
| In their positive panel, reindeer and moose are present, but no red deer samples were included. Since the rapid test sensitivity is not always the same for moose and reindeer (see results with TeSeE SAP Combi test), inclusion of red deer positive samples in the positive panel would have strengthened the presented results. Could the authors comment on this point or add some data regarding red deer? | The authors assert that comparison analyses with different screening and confirmatory tests would certainly have provided important information on positive red deer cases as well, but unfortunately, the amount of the only three confirmed cases of positive red deer is minimal and did not allow further study.  The first red deer case was identified with the TeSeE ELISA and the second and third cases with the HerdCheck ELISA. All were confirmed by TeSeE Western blot. |
| How did the authors calculate their cut-off values? This is not explained in the manuscript? | The authors did not report the cut-off calculation as this is reported in the instructions from the manufacturers of the tests; nevertheless this clarification is now reported in the manuscript. |
| Line 188, the authors mention that they used half of the Proteinase K concentration. Could the authors comment on this? | The authors used half of the test PK concentration as initial analysis performed in the laboratory yielded the best results in terms of PrP^Sc^ signal strength when reducing the concentration of this enzyme, without influencing the results for the negative animals. This change to the method, although reported in the materials and methods section of the manuscript, has now been further explained (Lines 203-205). |
| For a complete comprehension for the reader, some abbreviations would need to be explained (CC line 183, TMB line 184, RCF line 192, mAb line 198 (mAb is explained however later in the manuscript), ECL line 199, MP line 200, SAF line 248. | The authors have provided comprehensive explanations of various abbreviations in the text. |
| For homogeneity in their notation, the authors should choose between “g” and “RCF” in their materials and methods part when referring to centrifugation conditions, and also between “PK” (line 189 and “pK” (line 158) when referring to “proteinase K”. | The authors have conformed the different definitions of centrifugation steps with “g” and “PK” when referring to “proteinase K”. |
| The TBS composition is not mentioned (line 203). | The authors felt there was no need to specify the composition of this buffer, as it is commonly used in laboratories. |
| Some concentrations are given as “microg ml” instead of “microg per ml” or “microg ml-1” (lines 205 and 216). | The authors have revised the text accordingly. |
| The transfer conditions onto PVDF membranes are not mentioned (line 209) | The authors have reported in the text the transfer conditions. |
